# Flexible Development Programs for Antibacterial Drugs to Address Unmet Medical Needs 

**DOI:** 10.3201/eid3011.231416

**Published:** 2024-11

**Authors:** Mayurika Ghosh, Dmitri Iarikov, Xiaojing (Karen) Qi, Daniel Rubin, Simone Shurland, Avery Goodwin, Xiaohui Wei, Dakshina Chilukuri, Owen McMaster, Terry Miller, Peter Kim, Adam Sherwat

**Affiliations:** Center for Drug Evaluation and Research, US Food and Drug Administration, Silver Spring, Maryland, USA

**Keywords:** bacteria, antimicrobial resistance, carbapenem-resistant *Acinetobacter baumannii-calcoaceticus* complex, sulbactam, durlobactam, unmet medical need, antibacterial drug development, US Food and Drug Administration, United States

## Abstract

The US Food and Drug Administration recognizes the unmet medical need for antibacterial drugs to treat serious bacterial diseases caused by resistant pathogens for which effective therapies are limited or lacking. The agency also recognizes that designing and conducting clinical trials to assess the safety and efficacy of drugs to treat resistant infections is challenging, especially for drugs only active against a single or a few bacterial species, and that a more flexible development program might be appropriate. In this article, we discuss several regulatory considerations for flexible development programs for antibacterial drugs intended to meet an unmet medical need. As an example, we use the recent approval of sulbactam for injection and durlobactam for injection (XACDURO) for the treatment of hospital-acquired bacterial pneumonia and ventilator-associated bacterial pneumonia caused by susceptible isolates of *Acinetobacter baumannii-calcoaceticus* complex.

As described in guidance from the US Food and Drug Administration (FDA), the evaluation of antibacterial drugs for serious bacterial diseases is associated with several challenges, such as the need to promptly initiate empiric antibacterial therapy that might obscure effects of the investigational drug(s) in noninferiority trials and difficulties with obtaining informed consent in acutely ill patients (*1*). Developing drugs that are active against a single resistant bacterial species presents additional challenges. The number of patients with infections with a targeted resistant phenotype can be relatively small, thereby slowing accrual of evaluable patients into the trial. Lack of rapid diagnostics can result in uncertainty regarding bacterial etiology at the time of enrollment, thereby necessitating continuation of empiric antibacterial therapy pending confirmation of the pathogen of interest. In potentially polymicrobial infections, such as nosocomial pneumonia, continuing broad-spectrum antibacterial therapy might be needed throughout the study to provide coverage for bacterial species against which the study drug is not active. Ideally, the spectrum of activity of the concomitant antibacterial therapy should not overlap with the activity of the antibacterial drug being studied, but that difference is not always feasible. When overlapping antibacterial therapy is necessary, it can confound assessments not only of efficacy but also of safety of the investigational drug.

For serious bacterial diseases for which effective treatment options exist, the efficacy of an investigational drug can be established in a noninferiority trial. The clinical trial population should include persons whose illness severity and comorbidities reflect the patient population with unmet medical need to ensure safety and efficacy findings are generalizable (*1*). Only patients who have pathogens against which the control drug has antibacterial activity should be included in the efficacy analyses in noninferiority trials. Thus, a higher than anticipated prevalence of resistance to the selected comparator might require increasing the initially planned sample size.

## The Example of Sulbactam/Durlobactam

Infections caused by carbapenem-resistant (CR) *Acinetobacter baumannii-calcoaceticus* complex (ABC) constitute an area of high unmet medical need. They are associated with significant mortality rates, ranging from 38% to 76%; nosocomial pneumonia is the most common infection type (*2*–*5*). In 2020 in the United States, an estimated 7,500 cases of CR ABC infection occurred, resulting in 700 deaths (*6*). Therapies to treat CR ABC infections are limited because of multiple resistance mechanisms demonstrated by *A. baumannii*. In patients with hospital-acquired bacterial pneumonia (HABP) or ventilator-associated bacterial pneumonia (VABP) caused by a CR pathogen that is sensitive only to polymyxins, treatment with intravenous polymyxins (colistin or polymyxin B) is an option (*7*). However, current treatment guidelines acknowledge that no clear standard-of-care regimen exists for the treatment of CR ABC infections (*8*).

XACDURO (Innoviva Specialty Therapeutics, Inc., https://www.xacduro.com) is a combination of sulbactam and durlobactam that is administered intravenously (*9*). Sulbactam is an Ambler class A β-lactamase inhibitor; however, against *A. baumannii*, sulbactam exerts its effect by binding to penicillin-binding proteins, thereby inhibiting cell-wall biosynthesis (*10*). An increasing number of isolates possess >1 β-lactamases that inactivate sulbactam (*11*). Durlobactam is a novel non–β‐lactam β‐lactamase inhibitor inactivating several serine β‐lactamases expressed by *Acinetobacter* that degrade sulbactam including those of Ambler classes A, C, and D. Durlobactam does not have intrinsic antibacterial activity against *A. baumannii*.

Approval of sulbactam/durlobactam relied primarily on a single phase 3, randomized, independent assessor–blinded, active-controlled noninferiority study in adults with HABP or VABP caused by CR ABC in which sulbactam/durlobactam was compared with colistin (*12*). A noninferiority margin of 20% was used in the clinical trial (*13*). Both treatment arms also received imipenem/cilastatin as background therapy for potential HABP/VABP pathogens other than CR ABC. The primary efficacy endpoint for the study was 28-day all-cause mortality.

A total of 177 patients with documented ABC infections were randomized and received study drug (91 in the sulbactam/durlobactam group and 86 in the colistin group); 128 of 177 patients were found to have CR ABC infection susceptible to sulbactam/durlobactam and colistin. Among the 128 patients, 125 patients did not withdraw consent before assessment of survival status at day 28 and were included in the efficacy analyses. Sulbactam/durlobactam was found to be noninferior to colistin for the 28-day all-cause mortality primary endpoint (sulbactam/durlobactam 19% [12/63] vs. colistin 32.3% [20/62]; treatment difference: −13.2% [95% CI −30.0% to 3.5%]).

In many situations, FDA requires 2 adequate and well-controlled trials to establish effectiveness. Under certain circumstances, FDA can conclude that 1 adequate and well-controlled trial plus confirmatory evidence is sufficient to establish effectiveness. Factors considered when determining whether reliance on a single trial is appropriate include the persuasiveness of the single trial and the seriousness of the disease, particularly when an unmet medical need exists (*14*,*15*). Confirmatory evidence in the sulbactam/durlobactam development program was provided by in vitro studies, which were complemented by animal studies in murine neutropenic thigh abscess and lung infection models that established pharmacokinetic/pharmacodynamic targets for sulbactam/durlobactam combination and demonstrated that durlobactam restored sulbactam bactericidal activity against *A. baumannii* isolates producing serine β-lactamases (*15*,*16*).

Sulbactam and durlobactam pharmacokinetic data from phase 1, 2, and 3 studies were used for population pharmacokinetic modeling and probability of pharmacokinetic/pharmacodynamic target attainment analyses to inform the sulbactam/durlobactam dosage regimen and susceptibility breakpoints. Pharmacokinetic/pharmacodynamic target attainment results were also provided for drug exposures in epithelial lining fluid where animal and human data on the lung penetration for sulbactam and durlobactam were considered. Sulbactam and durlobactam pharmacokinetics were also evaluated in patients with altered renal function, which informed the dosage in such patients.

For a drug that is the subject of a more flexible development program, a safety database should generally include ≈300 persons at the dose and duration of therapy proposed for marketing. The safety database for the sulbactam/durlobactam new drug application included 158 participants who received sulbactam and durlobactam at the proposed dose and duration, including a phase 2 study in complicated urinary tract infections and the phase 3 study in HABP/VABP. The safety of durlobactam was also evaluated in 6 phase 1 studies in which 123 patients received durlobactam and 72 patients received sulbactam and durlobactam combinations.

No unexpected safety signals were identified in clinical studies during the development program, and the safety profile of sulbactam/durlobactam appeared consistent with other β-lactam/β-lactamase inhibitors. In the phase 3 trial, the incidence of acute kidney injury was lower in the sulbactam/durlobactam group (6%) than in the colistin group (36%) (*13*). No specific safety concerns were identified in nonclinical studies of sulbactam and durlobactam. In addition, clinical experience with sulbactam, which is approved in the United States in combination with ampicillin, was considered. Thus, although the safety database of the sulbactam/durlobactam new drug application was relatively limited and some uncertainties remained, because this combination addresses an unmet medical need for a serious disease and demonstrated efficacy on the basis of an all-cause mortality endpoint, FDA determined that the database was adequate to inform the risk-benefit assessment. During an Antimicrobial Drugs Advisory Committee meeting on April 17, 2023, the Committee unanimously voted that the overall benefit‐risk assessment was favorable for the use of sulbactam/durlobactam for the treatment of patients with HABP and VABP caused by susceptible strains of ABC organisms (*17*).

When the approval standard has been met but uncertainties remain about findings of a potentially serious risk, the FDA might determine that a postmarketing study is needed to further characterize the risk. In the case of sulbactam/durlobactam, a postmarketing requirement to conduct a single-arm, open-label, prospective, observational study to assess safety, including the risk for hypersensitivity reactions, in patients with ABC infection was issued at the time of approval to collect additional safety data.

In conclusion, FDA recognizes that challenges to developing antibacterial drugs to treat serious and life-threatening infections exist, especially for infections caused by highly drug-resistant pathogens for which treatment options are limited. The development program for sulbactam/durlobactam illustrates the successful use of a flexible development program for an antibacterial drug to address an unmet medical need.

## References

[1] US Food and Drug Administration. Antibacterial therapies for patients with an unmet medical need for the treatment of serious bacterial diseases—questions and answers (revision 1) guidance for industry, May 2022 [cited 2023 Jul 10]. https://www.fda.gov/media/158589/download

[2] Aydemir H, Akduman D, Piskin N, Comert F, Horuz E, Terzi A, et al. Colistin vs. the combination of colistin and rifampicin for the treatment of carbapenem-resistant *Acinetobacter baumannii* ventilator-associated pneumonia. Epidemiol Infect. 2013;141:1214–22. 10.1017/S095026881200194X 22954403 PMC9151808

[3] Lemos EV, de la Hoz FP, Einarson TR, McGhan WF, Quevedo E, Castañeda C, et al. Carbapenem resistance and mortality in patients with *Acinetobacter baumannii* infection: systematic review and meta-analysis. Clin Microbiol Infect. 2014;20:416–23. 10.1111/1469-0691.12363 24131374

[4] Paul M, Daikos GL, Durante-Mangoni E, Yahav D, Carmeli Y, Benattar YD, et al. Colistin alone versus colistin plus meropenem for treatment of severe infections caused by carbapenem-resistant Gram-negative bacteria: an open-label, randomised controlled trial. Lancet Infect Dis. 2018;18:391–400. 10.1016/S1473-3099(18)30099-9 29456043

[5] Weiner-Lastinger LM, Abner S, Edwards JR, Kallen AJ, Karlsson M, Magill SS, et al. Antimicrobial-resistant pathogens associated with adult healthcare-associated infections: Summary of data reported to the National Healthcare Safety Network, 2015-2017. Infect Control Hosp Epidemiol. 2020;41:1–18. 10.1017/ice.2019.296 31767041 PMC8276252

[6] Castanheira M, Mendes RE, Gales AC. Global epidemiology and mechanisms of resistance of *Acinetobacter baumannii-calcoaceticus* complex. Clin Infect Dis. 2023;76(Suppl 2):S166–78. 10.1093/cid/ciad109 37125466 PMC10150277

[7] Kalil AC, Metersky ML, Klompas M, Muscedere J, Sweeney DA, Palmer LB, et al. Management of Adults With Hospital-acquired and Ventilator-associated Pneumonia: 2016 Clinical Practice Guidelines by the Infectious Diseases Society of America and the American Thoracic Society. Clin Infect Dis. 2016;63:e61–111. 10.1093/cid/ciw353 27418577 PMC4981759

[8] Tamma PD, Aitken SL, Bonomo RA, Mathers AJ, van Duin D, Clancy CJ. Infectious Diseases Society of America 2023 guidance on the treatment of antimicrobial resistant Gram-negative infections. Clin Infect Dis. 2023;ciad428; Epub ahead of print. 10.1093/cid/ciad428 37463564

[9] US Food and Drug Administration. XACDURO (sulbactam for injection; durlobactam for injection) prescribing information. May 2023 [cited 2023 Jul 10]. https://www.accessdata.fda.gov/drugsatfda_docs/label/2023/216974Orig1s000Correctedlbl.pdf

[10] Penwell WF, Shapiro AB, Giacobbe RA, Gu RF, Gao N, Thresher J, et al. Molecular mechanisms of sulbactam antibacterial activity and resistance determinants in *Acinetobacter baumannii.* Antimicrob Agents Chemother. 2015;59:1680–9. 10.1128/AAC.04808-14 25561334 PMC4325763

[11] Shapiro AB, Moussa SH, McLeod SM, Durand-Réville T, Miller AA. Durlobactam, a new diazabicyclooctane β-lactamase inhibitor for the treatment of *Acinetobacter* infections in combination with sulbactam. Front Microbiol. 2021;12:709974. 10.3389/fmicb.2021.709974 34349751 PMC8328114

[12] Kaye KS, Shorr AF, Wunderink RG, Du B, Poirier GE, Rana K, et al. Efficacy and safety of sulbactam-durlobactam versus colistin for the treatment of patients with serious infections caused by *Acinetobacter baumannii-calcoaceticus* complex: a multicentre, randomised, active-controlled, phase 3, non-inferiority clinical trial (ATTACK). Lancet Infect Dis. 2023;23:1072–84. 10.1016/S1473-3099(23)00184-6 37182534

[13] US Food and Drug Administration. Sulbactam-durlobactam new drug application. Integrated review, application number: 216974Orig1s000 [cited 2024 Apr 29]. https://www.accessdata.fda.gov/drugsatfda_docs/nda/2023/216974Orig1s000IntegratedR.pdf

[14] US Food and Drug Administration. Demonstrating substantial evidence of effectiveness for human drug and biological products. Draft guidance for industry, December 2019 [cited 2023 Jul 10]. https://www.fda.gov/regulatory-information/search-fda-guidance-documents/demonstrating-substantial-evidence-effectiveness-human-drug-and-biological-products

[15] US Food and Drug Administration. Demonstrating substantial evidence of effectiveness with one adequate and well-controlled clinical investigation and confirmatory evidence, September 2023 [cited 2024 Apr 29. https://www.fda.gov/regulatory-information/search-fda-guidance-documents/demonstrating-substantial-evidence-effectiveness-one-adequate-and-well-controlled-clinical

[16] US Food and Drug Administration. Codevelopment of two or more new investigational drugs for use in combination, June 2013 [2024 Apr 29]. https://www.fda.gov/regulatory-information/search-fda-guidance-documents/codevelopment-two-or-more-new-investigational-drugs-use-combination

[17] Antimicrobial Drugs Advisory Committee. Updated information: April 17, 2023: meeting of the Antimicrobial Drugs Advisory Committee meeting announcement [cited 2024 Aug 30]. https://www.fda.gov/advisory-committees/advisory-committee-calendar/updated-information-april-17-2023-meeting-antimicrobial-drugs-advisory-committee-meeting#event-materials

